# The Book World of Medicine and Science

**Published:** 1906-01-13

**Authors:** 


### The Book World of Medicine and Science.

Notes on General Practice. By S. M. Hebblethwaite, M.D. (London: The Scientific Press, 1905. Pp. 78. Price 3s. 6d. net.)

It has doubtless occurred to many others, as well as to the author of these Notes, that it is matter for regret that the mass of information about disease possessed by many general practitioners cannot be more widely diffused through the medical profession. That such information exists, the excellent practical work done by many general practitioners in all parts of the country furnishes sufficient proof, and who amongst us has not learned much for which text-books might be searched in vain from such a man. But unfortunately these are not the men who contribute most to medical literature. The little book before us makes no pretence to completeness, but it indicates some of the many directions in which the general practitioner might add considerably to our knowledge. Strong in the belief that the same mistakes are made by succeeding generations of practitioners, Dr. Hebblethwaite has set himself to guard the steps of the unwary by a practical description of some of the more frequent pitfalls met with in practice. His advice is in all cases, whether medical or surgical, whether as to diagnosis or treatment, characterised by method and a sound common sense; while his notes on perforative peritonitis, rheumatism in children, caries of the cervical spine, and retention of urine from retroflection of the gravid uterus may be specially singled out as being worthy of remembrance. An even better safeguard against error is the spirit of intelligent interest and trained observation with which it is evident that the author himself approaches the bedsides of his patients.

Counsels and Ideals from the Writings of William Osler. By C. N. B. Camac (Oxford: Henry Frowde. Pp. 277. Price 4s.)

This is a neat little volume compiled from the writings, addresses, and lectures of William Osler. Many of the passages are so full of interest that one regrets they are so short; yet the thoughts expressed supply much food to the mind and stimulate and increase the reader's own thinking and reasoning powers. The extracts are grouped under different headings, such as "Exemplary Characters in Medicine;" "The Practical in Medicine," "Silence and Self-control," "Charity and Fraternity in Medicine," "Value of Travel," "Work." Each extract has a reference number to the original which, in many instances, readers may desire to read throughout. This book may serve to pilot a man or woman in the early years, and those who are embarked on the full stream of life will find much that is of value in forming and developing new traits of character.

Surgical Instruments and Appliances Used in Operations. By Harold Burrows, M.B., F.R.C.S. (London: The Scientific Press, 1905. Pp. 96. Illustrated. Price 1s. 6d. net.)

This book may be best described in the words of its subtitle as an "illustrated and classified list with explanatory notes," intended to guide those on whom the duty of making arrangements for a surgical operation may fall. The method adopted is the provision of lists of instruments for the various operations, each of the appliances being illustrated from the catalogue of a well-known instrument maker. In this way the veriest tyro, whether probationer or student, may be depended on to find what is required for any operation, and probably this is the class of assistant for which the book is intended. For the surgeon such works are of little use, and though instruments are sometimes forgotten, such omissions will be minimised if the rule be adopted of mentally going over the operation, laying aside each instrument in the order in which it will be used, beginning with the anæsthetic and finishing with the necessary dressings. Some practical directions as to the arrangements necessary in preparing for an operation in a private house should prove useful to nurses new to the requirements of practice without the conveniences and appliances of a hospital.

The Advertiser's A B C Press Directory. (Published by T. B. Browne, Limited, 163 Queen Victoria Street, E.C. Price 10s. 6d.)

contains nearly 1,100 pages, and is admirably printed on art paper. Among the more important features of the book are lists of circulations of London and provincial papers, London offices and representatives of provincial papers. A full directory of the Press of the United Kingdom, a directory of the Press of the British Colonies and India, and selected lists of European and African papers. The book is very complete and accurate, and has long been recognised as the standard work of reference on such matters by advertisers, journalists, and business men.

Willing's Press Guide, 1906. (125 Strand, London, W.C. Price 1s.)

We welcome the thirty-third annual issue of this most handy little Press guide. The new edition appears to have been thoroughly revised and brought up to date, and combines all the advantages of arrangement, etc., that has long made its predecessors so useful and handy of reference.

Practical Advertising, 1905-6. (Published by Messrs. Mather and Crowther, 10, 11, 12 New Bridge Street, E.C.)

The new issue of this Press Directory contains classified lists of newspapers, magazines, and periodicals, which are both complete and accurate. It also contains some excellent examples of modern advertisement printing, and should prove useful to those for whom the work is intended.

